# Evaluation of an Artificial Intelligence-Based Tool and a Universal Low-Cost Robotized Microscope for the Automated Diagnosis of Malaria

**DOI:** 10.3390/ijerph22010047

**Published:** 2024-12-31

**Authors:** Carles Rubio Maturana, Allisson Dantas de Oliveira, Francesc Zarzuela, Alejandro Mediavilla, Patricia Martínez-Vallejo, Aroa Silgado, Lidia Goterris, Marc Muixí, Alberto Abelló, Anna Veiga, Daniel López-Codina, Elena Sulleiro, Elisa Sayrol, Joan Joseph-Munné

**Affiliations:** 1 Microbiology Department, Vall d’Hebron University Hospital, Vall d’Hebron Research Institute (VHIR), 08035 Barcelona, Spain; carles.rubio@vhir.org (C.R.M.); francesc.zarzuela@vallhebron.cat (F.Z.); alejandro.mediavilla@vhir.org (A.M.); patricia.martinez@vhir.org (P.M.-V.); aroa.silgado@vhir.org (A.S.); lidia.goterris@vallhebron.cat (L.G.); marc.muixi@vallhebron.cat (M.M.); 2 Department of Microbiology and Genetics, Universitat Autònoma de Barcelona (UAB), 08193 Barcelona, Spain; 3 Computational Biology and Complex Systems Group, Physics Department, Universitat Politècnica de Catalunya (UPC), 08860 Castelldefels, Spain; allisson.dantas@upc.edu (A.D.d.O.); daniel.lopez-codina@upc.edu (D.L.-C.); 4 Centro de Investigación Biomédica en Red Enfermedades Infecciosas (CIBERINFEC), Instituto de Salud Carlos III, 28029 Madrid, Spain; 5 Database Technologies and Information Management Group, Service and Information Systems Engineering Department, Universitat Politècnica de Catalunya (UPC), 08034 Barcelona, Spain; alberto.abello@upc.edu; 6 Probitas Foundation, 08022 Barcelona, Spain; ana.veiga@fundacionprobitas.org; 7 Tecnocampus, Universitat Pompeu Fabra, 08302 Mataró, Spain

**Keywords:** artificial intelligence, malaria, automated diagnosis, tropical medicine, *Plasmodium*, point-of-care, infectious diseases

## Abstract

The gold standard diagnosis for malaria is the microscopic visualization of blood smears to identify *Plasmodium* parasites, although it is an expert-dependent technique and could trigger diagnostic errors. Artificial intelligence (AI) tools based on digital image analysis were postulated as a suitable supportive alternative for automated malaria diagnosis. A diagnostic evaluation of the *iMAGING* AI-based system was conducted in the reference laboratory of the International Health Unit Drassanes-Vall d’Hebron in Barcelona, Spain. *iMAGING* is an automated device for the diagnosis of malaria by using artificial intelligence image analysis tools and a robotized microscope. A total of 54 Giemsa-stained thick blood smear samples from travelers and migrants coming from endemic areas were employed and analyzed to determine the presence/absence of *Plasmodium* parasites. AI diagnostic results were compared with expert light microscopy gold standard method results. The AI system shows 81.25% sensitivity and 92.11% specificity when compared with the conventional light microscopy gold standard method. Overall, 48/54 (88.89%) samples were correctly identified [13/16 (81.25%) as positives and 35/38 (92.11%) as negatives]. The mean time of the AI system to determine a positive malaria diagnosis was 3 min and 48 s, with an average of 7.38 FoV analyzed per sample. Statistical analyses showed the Kappa Index = 0.721, demonstrating a satisfactory correlation between the gold standard diagnostic method and *iMAGING* results. The AI system demonstrated reliable results for malaria diagnosis in a reference laboratory in Barcelona. Validation in malaria-endemic regions will be the next step to evaluate its potential in resource-poor settings.

## 1. Introduction

Malaria is a parasitic disease caused by *Plasmodium* spp. with a high prevlence in tropical regions worldwide and transmitted through the bites of *Anopheles* female mosquitoes. The five *Plasmodium* species that cause malaria infection in humans are *Plasmodium falciparum*, *P. vivax*, *P. ovale*, *P. malariae,* and *P. knowlesi*. Regarding World Health Organization (WHO) data, globally, in 2022, there were 249 million malaria cases and 608,000 deaths [1]. Most of the deaths are caused by *P. falciparum*, mainly in children under five years of age from resource-poor setting areas of the Sub-Saharan Africa region. Therefore, malaria early diagnosis is crucial to decrease morbidity and mortality. The microscopic examination of thick and thin blood smear samples to determine the presence and species of *Plasmodium* parasites is the gold standard technique for malaria diagnosis. However, it is an expert-dependent methodology and could trigger diagnostic errors. As a supportive tool for microscopy examination, artificial intelligence (AI) methods are being postulated due to their easy handling. Novel image analysis techniques encompass Convolutional Neural Networks (CNNs), attention modules, and transformers, which all determine and identify objects of interest in digital images [2]. In addition, the robotization of the microscope stage and image autofocus would provide a fully automatic process. Low-cost robotized designs were proposed as suitable alternatives for conventional optical microscopy automation [3]. Moreover, several AI-based systems have been evaluated for the diagnosis of malaria and schistosomiasis in laboratories from endemic areas [4,5,6]. Most systems employ a smartphone device to acquire and analyze digital images or videos by CNN models in order to identify parasites or bacteria and perform a final diagnosis.

The *iMAGING* system is based on a robotized low-cost system consisting of 3D pieces and servo motors to automate a light optical microscope and a smartphone application responsible for acquiring the images and analyzing them by the You Only Look Once (YOLOv5) CNN pre-trained model. The development and design of the diagnostic system were already published in previous studies [7,8].

In this study, a preliminary diagnostic evaluation of the *iMAGING* system for the diagnosis of malaria in Giemsa-stained thick blood smear samples was performed. We evaluated the diagnostic sensitivity and specificity of the AI system in comparison with the gold standard methodology. Therefore, this study represents the initial step before the diagnostic validation in malaria-endemic settings.

## 2. Materials and Methods

### 2.1. Study Design

A diagnostic validation study was conducted using a collection of samples from travelers, Visit Friends and Relatives (VFR), and migrants coming from malaria-endemic areas (mainly Sub-Saharan Africa, South America, and Southeast Asia regions [9]) attending the International Health Unit Drassanes-Vall d’Hebron. The sample collection period was encompassed between 1 February 2023 and 30 November 2023. A total of 54 samples from 46 individuals were analyzed. Different samples from the same individual/patient were drawn at different times, before and after antimalarial drug treatment. Giemsa-stained thick blood smear samples were examined by conventional optical microscopy and were analyzed by the *iMAGING* system for malaria diagnosis. All *Plasmodium*-negative samples were also analyzed by RT-PCR (RealStar^®^ Malaria, Altona, Hamburg, Germany) for diagnosis confirmation. Positive samples were classified according to parasite density (<800 parasites/µL; 801–10,000 parasites/µL; or >10,000 parasites/µL). *Plasmodium* species information was also collected. All samples were retrospectively revised by a laboratory technician to ensure sample quality.

### 2.2. Sample Size

A total of 54 thick blood smear samples were estimated to be statistically representative of our study. The sample size was calculated from epidemiological data of the International Health Unit Drassanes-Vall d’Hebron laboratory, following statistics guidelines for sample size calculation with proportions [10]. The sample size equation requires population size, probability of a condition (malaria positive), confidence level, and accepted error. Since malaria is not endemic in Spain, an approximation with real clinical data was employed. The population size was taken to be the number of blood smears analyzed from patients with suspected malaria (84 patients from 1 January 2020 to 31 December 2020), with a prevalence of 10.71% in our center. The calculations were performed using a value of 95% confidence and a 5% accepted error.

### 2.3. Microscope Automation

Samples were analyzed using the *iMAGING* software version 1.0. smartphone application for *Plasmodium* parasite detection in thick blood smear samples. Sample scanning was automated by the robotization of a conventional optical microscope (Olympus CH-2, Olympus, Feasterville-Trevose, PA, USA) with polylactic acid (PLA) 3D pieces. Microscope stage movements and autofocus issues were automated by three 9G low-cost servo motors, one for each axis, *X*-*Y* and *Z* (focus). All the system was controlled by an MKR Wifi 1010 Arduino controller (Arduino, Monza, Italy), connected by Bluetooth (BLE) to the smartphone device (Xiaomi Redmi 10, Xiaomi Corporation, Beijing, China). The robotized microscope system design has been published in open-access format [8]. Samples were analyzed through a snake-like movement in order to correctly visualize the thick blood smear sample. Autofocus analysis was performed via the Laplacian Variance algorithm in each Field of View (FoV).

### 2.4. Image Analysis and Diagnostic Algorithm

Digital images were acquired through the ocular lens of the robotized microscope with a Xiaomi Redmi 10C smartphone device (Camera: 720 × 1650 pixels resolution; Dual 50 MP, f/1.8, 26 mm (wide), PDAF 2 MP, f/2.4, (depth), HDR). A minimum of 30 MP resolution camera is required for image analysis. A two-step process was designed for the analysis of the images via the YOLOv5 CNN model to obtain more reliable results. YOLOv5 is a state-of-the-art CNN algorithm with high-performance results in multiple image analysis applications in terms of accuracy and speed. The detection of objects (parasites and cells) in digital images is a suitable task for that kind of model. Previous studies also demonstrate that the YOLOv5 model is the best option for the *iMAGING* diagnostic system for thick blood smear image analysis [7].

First, a live video of each FoV was recorded and analyzed using the Laplacian Variance algorithm. The captured video frame with the highest value of Laplacian Variance was analyzed by the neural network model YOLOv5s (smartphone) integrated into the *iMAGING* smartphone application. The YOLOv5s confidence threshold value was set to 0.25 for leukocyte and *Plasmodium* trophozoite detection via a smartphone device. This screening procedure was repeated until the identification of 15 possible parasites or the observation of 100 FoV, following data from previous studies.

**Figure 1:**
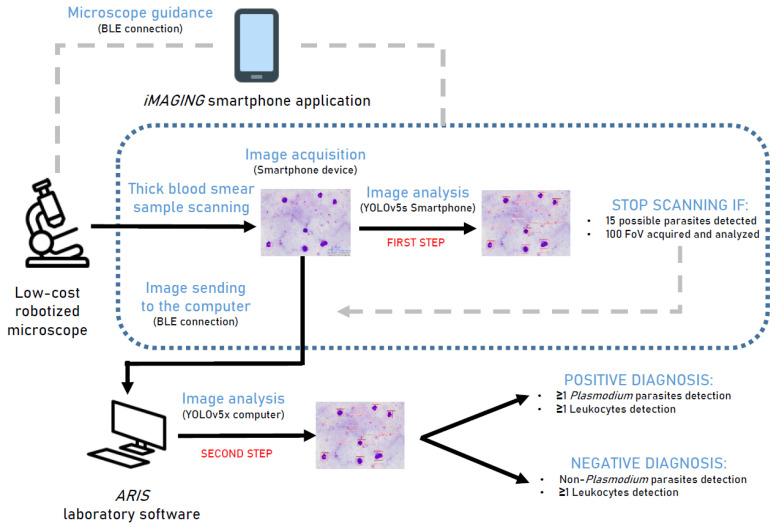
Diagnostic flowchart of the *iMAGING* smartphone application software and ARIS software for *Plasmodium* parasites detection in thick blood smear samples.

Secondly, analyzed images were sent by BLE connection to ARIS software (computer device) for diagnosis confirmation. YOLOv5x (computer) confidence threshold value was set to 0.25 (iMAGING) and 0.50 (iMAGINGv2) for leukocyte and *Plasmodium* trophozoites detection via a computer device. The variation in the threshold value (0.25 or 0.50) allows us to evaluate the diagnostic results of the CNN according to the level of restriction for object detection. Leukocyte detection allows us to confirm that the sample is correctly stained and serves as a quality control. If a single *Plasmodium* parasite was detected with the YOLOv5x model, the sample was considered positive. Conversely, if parasites were not detected in the analyzed images, the sample was considered negative for *Plasmodium* infection. The YOLOv5 metrics performance has already been published in previous studies by our research group [7]. A diagnostic flowchart is represented in Figure 1.

### 2.5. Statistical Analysis

*iMAGING* diagnostic results were compared with the reference gold standard diagnostic method (conventional optical microscopy). SPSS (version 27) and Excel 2016 were employed for statistical analysis. Diagnostic performance parameters for the *iMAGING* system were calculated: sensitivity, specificity, positive predictive value (PPV), negative predictive value (NPV), parasitemia densities, and time of analysis.

## 3. Results

### 3.1. Diagnostic Accuracy of the iMAGING System Compared with Light Optical Microscopy

A total of 54 thick blood smear samples from 46 individuals were collected and evaluated by optical microscopy (gold standard) and *iMAGING* (AI system). Among the 46 participants of this study, 35/46 (76%) were male and 11/46 (24%) female. Their mean age was 32 years old (y.o.) [Interquartile range (IQR): 21–42 y.o.], and 24/46 (52.2%) were migrants, 11/46 (23.9%) VFR, and 11/46 (23.9%) travelers. Samples analyzed by optical microscopy show 16/54 (29.6%) positive and 38/54 (70.3%) negative for *Plasmodium* infection. The positive samples showed parasitemia ranges between 80 to >10,000 parasites/µL of blood [7/16 (43.75%) show <800 parasites/µL; 4/16 (25.0%) show 801–10,000 parasites/µL; and 5/16 (31.25%) show >10,000 parasites/µL]. Samples were predominantly 10/16 (62.5%) *P. falciparum* infections. A total of 4/16 (25%) samples were *P. vivax*/*P. ovale* infections, and 2/16 (12.5%) were *P. malariae* infections. All negative samples, 38/38 (100%), were screened by RT-PCR (RealStar^®^ Malaria, Altona), showing negative results for *Plasmodium* infection. When compared to light microscopy, the *iMAGING* system demonstrated an 81.25% sensitivity (CI 95%: 53.69, 95.03), 92.11% specificity (CI 95%: 77.52, 97.94), 81.25% PPV (CI 95%: 53.69, 95.03), and 92.11% NPV (CI 95%: 77.52, 97.94). Overall, the AI system demonstrates an 88.89% accuracy (48/54). The *iMAGING* system correctly identified 48/54 samples, with three false-positive (3/16) and three false-negative results (3/38). Results are represented in Table 1. The Kappa value (CI 95%) = 0.721 (0.511, 0.931) demonstrated a relatively strong correlation.

### 3.2. Parasite Density and Plasmodium Species

**Figure 2:**
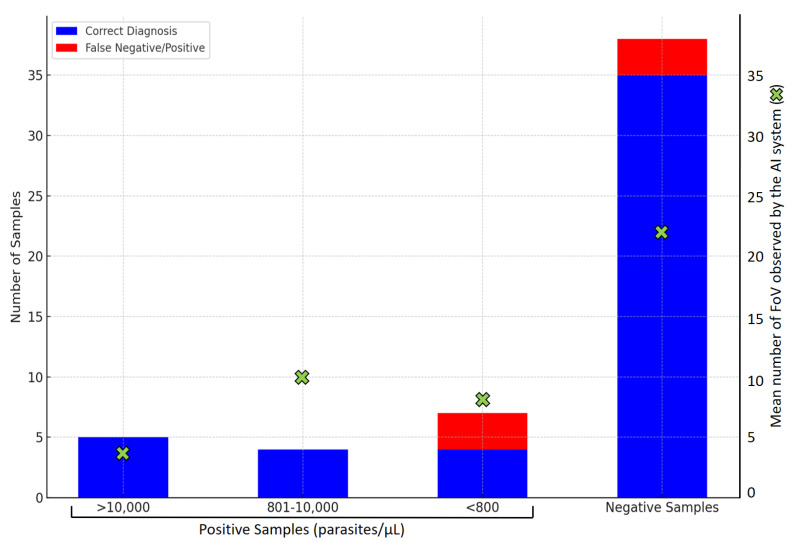
Diagnosis accuracy results of the *iMAGING* system. Positive samples were divided by parasite densities (>10,000 parasites/µL, 801–10,000 parasites/µL, and <800 parasites/µL). Green crosses represent the mean number of Fields of View (FoV) observed for each sample category.

In detail, diagnostic results comparisons are represented in Table A1. Regarding positive samples (*n* = 16), 13/16 (81.25%) were correctly considered positive for *Plasmodium* infection by the *iMAGING* device. False-positive and false-negative results were analyzed in detail to avoid diagnostic errors. Parasite density is a crucial feature for malaria diagnosis, and discordant results were interpreted. Regarding positive samples, 5/5 (100%) with >10,000 parasites/µL, 4/4 (100%) with 801–10,000 parasites/µL, and 4/7 (57.14%) with <800 parasites/µL were correctly diagnosed by the *iMAGING* system. A high percentage of false-negative results (3/7, 42.85%) were reported in low-parasitized samples (<800 parasites/µL), representing 5.55% (3/54) of all analyzed samples. Regarding negative samples (*n* = 38), 35/38 (92.10%) were correctly considered negative for *Plasmodium* infection by the *iMAGING* device. Overall, there were 3/38 (7.89%) false-positive results, mainly due to the high presence of artifact samples because of the Giemsa staining procedure. Diagnostic evaluation results are summarized in Figure 2. In terms of *Plasmodium* species, the system is not able to distinguish them, although results were analyzed regarding diagnostic positivity/negativity. In summary, 8/10 (80%) of *P. falciparum* samples, 3/4 (75%) of *P. vivax/P. ovale*, and 2/2 (100%) of *P. malariae* were correctly considered positive *Plasmodium* infections.

### 3.3. iMAGING System Scanning Performance

The mean number of FoV observed was 7.38 FoV for positive samples and 21.86 FoV for negative samples. Considering parasite density, a mean number of 4.4 FoV was observed in samples with >10,000 parasites/µL, 10 FoV in samples with 801–10,000 parasites/µL, and 8.5 FoV in samples with <800 parasites/µL (Figure 2). The number of observed FoV was considerably higher in negative samples than in positive samples (*p* < 0.01) due to the stop scanning criteria (15 possible parasites detected or 100 FoV observed).

In terms of time of analysis, the *iMAGING* system demonstrates a mean time of scanning of 3 min and 48 s for positive samples (*n* = 16). Negative samples (*n* = 38) were scanned in a mean time of 5 min and 5 s, considering that in most occasions the stopping criteria of 100 FoV observed was not reached.

## 4. Discussion

The *iMAGING* AI-based diagnostic system has demonstrated reliable results for malaria diagnosis in non-endemic settings. A fully automated procedure in a smartphone application and a low-cost robotized microscope confer the system the suitable features to complement traditional microscopy diagnosis. Diagnostic evaluation results of sensitivity and specificity by using the *iMAGING* AI system are in concordance with other similar studies [6,11,12]. A similar system, called EasyScan GO, based on AI malaria diagnosis with deep neural networks, demonstrated a range of 88.0–91.1% sensitivity and 75.6–89.0% specificity [6,11,12]. In terms of specificity, our system demonstrated more optimal results, although, in terms of sensitivity, EasyScan GO outperforms *iMAGING*. Other similar devices, such as miLAB^TM^, an automated microscope for the detection of *Plasmodium* parasites in thin blood smears, were evaluated for detecting the parasites in samples from symptomatic patients at point-of-care in Sudan [12]. Accuracy results demonstrate a 91.1% sensitivity and 66.7% specificity in the automated mode, although specificity increased to 96.2% with operator intervention [12]. The cited devices demonstrate the applicability of automated tools, such as *iMAGING*, as supportive tools for point-of-care malaria diagnosis. However, AI-systems comparison should be pursued with the same samples and in the same laboratory conditions in order to reliably compare the diagnostic potential of each device.

The two-step diagnostic procedure allows the system to avoid possible false-positive results by the YOLOv5s model. YOLOv5s was executed by the smartphone device; therefore, its computational potential and performance were lower in comparison with YOLOv5x (computer). All sample scanning analyses were stopped by the ≥15 possible parasites detected criteria (Figure 1) and screened via the YOLOv5x model to discard false-positive results. However, less than 100 FoV were observed in each sample, causing negative results to depend on the reliability of the observed fields. Although the proposed methodology significantly reduces the time of analysis for each sample, equaling that of a microscopist.

Another determinant factor for the diagnostic evaluation is the previous development validation of the diagnostic system, as performed with the *iMAGING* system [3]. Neural network validation should be performed before a diagnostic validation. Convolutional Neural Network (CNN) performance is crucial to obtain a reliable diagnosis, although optimal CNN metrics are not sufficient to evaluate a diagnostic system, as they provide information about the accuracy of the detection of objects in digital images and not about the final diagnosis of the analyzed sample. Several studies demonstrate optimal CNN metrics for malaria detection in thick and thin blood smear samples [13,14,15]. However, diagnostic validations should be the next step in order to evaluate detection software as a supportive novel diagnostic tool. Artificial intelligence-based systems could be a suitable supportive tool for malaria diagnosis in non-endemic settings, although low parasite-density infections continue to challenge diagnostic accuracy [16]. The implementation of this type of technology in real clinical diagnostic conditions must be accompanied by microscopists, and the results should be interpreted by observing the digital images displayed by the system. In addition, we could avoid the number of false-positive and false-negative results due to errors in image analysis by means of CNNs.

This is the first diagnostic evaluation study of the *iMAGING* system. Therefore, further validation studies will be needed in reference laboratories and in the field. Preliminary results demonstrate that the *iMAGING* device could be a suitable supportive solution for the diagnosis of malaria in cases of medium and high *Plasmodium* parasite density samples and negative samples, although it should be supervised by laboratory technicians to detect low parasite densities. The diagnostic system algorithm could be improved by modifying the threshold value, by adding more digital images to our dataset, or by improving image quality [17,18]. These strategies could be the future steps for the *iMAGING* validation in non-endemic settings.

Some of the challenges of our study were (i) to increase the sample size (*n* = 54) employed for diagnostic evaluation, (ii) the non-identification by the AI system of *Plasmodium* species, (iii) smartphone camera (image resolution), and (iv) the false-negative results in some of the low parasitized samples. Thus, further studies in endemic areas should be pursued in order to evaluate the robustness of the AI-based system diagnostics in other laboratories with a larger number of patients and samples and with low parasite densities. Retraining AI algorithms and improving the smartphone camera resolution, or the 3D pieces of the device, could also positively improve image analysis. Moreover, Giemsa staining, laboratory infrastructure, and microscope lenses could affect the image acquisition procedure and, therefore, the final diagnosis [3]. *Plasmodium* identification models should also be integrated into the system to perform a complete malaria diagnosis, as published in other similar studies [19,20]. The availability of electricity and the diagnostic protocols of each country or region can also be significant when implementing this type of device.

## 5. Conclusions

To conclude, the *iMAGING* system has demonstrated a reliable diagnostic correlation with the gold standard methodology. The improvement of the system via the retraining of CNN models and the validation in other laboratory settings are needed to optimize the system, especially for low-parasitized samples. Furthermore, the training of microscopists remains a global priority, and in order not to lose awareness, strategies could be generated based on accessible digital tools. We believe that this novel diagnostic system will join the global effort to fight against malaria and poverty-related diseases, breaking the digital gap between the north and the south.

## Figures and Tables

**Table 1:** Diagnostic evaluation of the *iMAGING* system versus light optical microscopy for the diagnosis of malaria in Giemsa-stained thick blood smear samples.

		**Light Microscopy** **(Gold Standard)**			**Sensitivity** **(CI 95%)**	**Specificity** **(CI 95%)**	**PPV** **(CI 95%)**	**NPV** **(CI 95%)**	**Kappa Value** **(CI 95%)**	***p*-Value**	**Mean Time of Analysis**	
***iMAGING*** **(AI system)**		(+)	(−)	Total	81.25 (53.69, 95.03)	92.11 (77.52, 97.94)	81.25 (53.69, 95.03)	92.11 (77.52, 97.94)	0.721 (0.511, 0.931) Significant *	0.05	Positive (*Plasmodium*+)	Negative (*Plasmodium*−)
	(+)	13	3 (FP)	16							3 min 48 s/sample	5 min 5 s */sample
	(−)	3 (FN)	35	38								
	Total	16	38	54								

PPV: positive predictive value; NPV: negative predictive value; AI: artificial intelligence; CI: confidence interval; FP: false positive; FN: false negative. * In most occasions, less than 100 Fields of View (FoV) were observed, significantly reducing the meantime of analysis. * Kappa Cohen’s value interpretation following Landis JR, Koch GG. The measurement of observer agreement for categorical data. Biometrics. 1977 criteria.

## Data Availability

The original contributions presented in the study are included in the article; further inquiries can be directed to the corresponding author.

## Appendix A

**Table A1:** Comparison of the diagnostic performance of the *iMAGING* system with the 54 analyzed samples in comparison with the gold standard diagnosis (International Health Unit Vall d’Hebron-Drassanes). All negative samples were also confirmed with *RT-PCR (RealStar^®^ Malaria, Altona)*.

**Sample Codification**	**Light Microscopy** **(Reference Laboratory)**	**AI System** **(Number of FoV Observed)**
1	POSITIVE (*P. falciparum*; +10,000 parasites/µL)	POSITIVE (2)
2	POSITIVE (*P. falciparum*; +10,000 parasites/µL)	POSITIVE (3)
3	POSITIVE (*P. falciparum*; +160 parasites/µL)	NEGATIVE (26)
4	POSITIVE (*P. falciparum*; +160 parasites/µL)	POSITIVE (13)
5	POSITIVE (*P. falciparum*; +640 parasites/µL)	NEGATIVE (43)
6	POSITIVE (*P. falciparum*; +7600 parasites/µL)	POSITIVE (24)
7	NEGATIVE	NEGATIVE (45)
8	NEGATIVE	NEGATIVE (18)
9	NEGATIVE	NEGATIVE (8)
10	NEGATIVE	NEGATIVE (15)
11	NEGATIVE	NEGATIVE (16)
12	NEGATIVE	NEGATIVE (7)
13	NEGATIVE	NEGATIVE (9)
14	NEGATIVE	NEGATIVE (20)
15	NEGATIVE	NEGATIVE (17)
16	NEGATIVE	POSITIVE (33)
17	NEGATIVE	NEGATIVE (5)
18	NEGATIVE	NEGATIVE (22)
19	NEGATIVE	NEGATIVE (88)
20	NEGATIVE	POSITIVE (21)
21	NEGATIVE	POSITIVE (27)
22	POSITIVE (*P. vivax*; +640 parasites/µL)	NEGATIVE (32)
23	POSITIVE (*P. vivax*; +640 parasites/µL)	POSITIVE (12)
24	POSITIVE (*P. falciparum*; +10,000 parasites/µL)	POSITIVE (1)
25	POSITIVE (*P. falciparum*; +10,000 parasites/µL)	POSITIVE (14)
26	NEGATIVE	NEGATIVE (18)
27	NEGATIVE	NEGATIVE (6)
28	NEGATIVE	NEGATIVE (29)
29	NEGATIVE	NEGATIVE (31)
30	POSITIVE (*P. falciparum*; 80 parasites/µL)	POSITIVE (5)
31	POSITIVE (*P. falciparum*; 640 parasites/µL)	POSITIVE (4)
32	NEGATIVE	NEGATIVE (7)
33	NEGATIVE	NEGATIVE (7)
34	NEGATIVE	NEGATIVE (18)
35	NEGATIVE	NEGATIVE (17)
36	NEGATIVE	NEGATIVE (16)
37	NEGATIVE	NEGATIVE (93)
38	NEGATIVE	NEGATIVE (45)
39	NEGATIVE	NEGATIVE (22)
40	NEGATIVE	NEGATIVE (10)
41	NEGATIVE	NEGATIVE (15)
42	NEGATIVE	NEGATIVE (7)
43	NEGATIVE	NEGATIVE (21)
44	NEGATIVE	NEGATIVE (12)
45	POSITIVE (*P. ovale*; +10,000 parasites/µL)	POSITIVE (2)
46	POSITIVE (*P. ovale*; +8200 parasites/µL)	POSITIVE (6)
47	NEGATIVE	NEGATIVE (53)
48	NEGATIVE	NEGATIVE (36)
49	NEGATIVE	NEGATIVE (9)
50	NEGATIVE	NEGATIVE (10)
51	POSITIVE (*P. malariae*; +5600 parasites/µL)	POSITIVE (6)
52	POSITIVE (*P. malariae*; +5400 parasites/µL)	POSITIVE (4)
53	NEGATIVE	NEGATIVE (9)
54	NEGATIVE	NEGATIVE (4)
